# Surgical Correction of a Severe Bilateral Cleft Foot Using a Diamond Incision and Cannulated Screw Fixation: A Case Report and Literature Review

**DOI:** 10.7759/cureus.98577

**Published:** 2025-12-06

**Authors:** Hassan H Alzein, Hadi Mouslem, Ahmad A El Lakis, Ahmad Zahwi, Youssef Daher

**Affiliations:** 1 Department of Orthopaedics and Traumatology, Lebanese University Faculty of Medicine, Beirut, LBN; 2 Department of Internal Medicine, American University of Beirut, Beirut, LBN

**Keywords:** cleft foot syndrome, developmental pediatrics, foot surgery techniques, pediatric case, surgical skills day

## Abstract

Cleft foot is a rare congenital deformity, characterized by the absence of one or more central rays of the foot and presenting with a wide spectrum of clinical and anatomical variations. Surgical intervention aims to improve this deformity on three levels: functional rehabilitation, cosmetic appearance, and the ability to wear standard footwear.

This case report describes the successful surgical management of a severe bilateral cleft foot (Blauth and Borisch Type III and Abraham Type II). The technique involved a diamond-shaped incision for cleft closure, cannulated screw fixation to narrow the forefoot, and correction of associated deformities. At one-year follow-up, the patient demonstrated pain-free ambulation, the ability to wear off-the-shelf standard footwear like peers, and satisfactory aesthetic results. This report highlights the importance of tailored surgical planning in managing the diverse variants of cleft foot and contributes to the growing literature by presenting a new, modified technique addressing its reconstructive challenges.

## Introduction

Cleft foot, also referred to as split foot, ectrodactyly, or “lobster claw,” is a rare congenital anomaly characterized by the deficiency of the central rays of the foot, corresponding to the second, third, and fourth metatarsals and their phalanges [1,2]. The severity of this deficiency can vary from partial shortening to the complete absence of these structures [2]. The cleft typically affects structures along the midline, usually beginning at the second or third ray and extending both longitudinally (from distal to proximal) and transversely (from tibial to fibular side). As a result, the defect is always more pronounced distally and generally spares the first and fifth metatarsals [1,3]. This condition is often bilateral and frequently occurs as part of a syndrome in association with other congenital anomalies, such as syndactyly [2,4].

The underlying cause of cleft foot is attributed to inadequate activity of the medial apical ectodermal ridge (AER), a key signaling center in limb bud development. This deficit leads to decreased cell proliferation and abnormal foot morphogenesis. This condition is most commonly inherited in an autosomal dominant pattern, with reduced penetrance, although autosomal recessive, X-linked, and even sporadic cases have also been reported. Cleft foot was first reported in South Africa in 1770, and the current prevalence is estimated by some authors to be approximately one in a million live births [4].

Given its rarity, no standardized treatment guidelines currently exist, and surgical management is typically individualized. Most reported cases have utilized techniques adapted from hand surgery with the primary goals of improving footwear compatibility, facilitating normal gait, and enhancing cosmetic appearance [1,2,4].

We present a clinical case of bilateral cleft foot that was treated surgically, with an excellent functional and aesthetic outcome. The severity of the deformity presented a unique clinical challenge, requiring meticulous preoperative planning and adaptation of the surgical technique to overcome the technical and reconstructive difficulties. The objective of this report is to describe the surgical approach, review the relevant literature, and outline the challenges and limitations encountered.

## Case presentation

**Figure 1:**
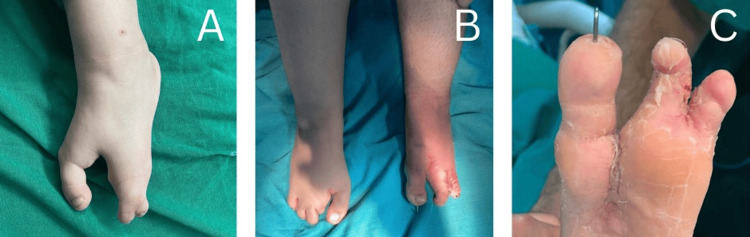
Comparison of preoperative and postoperative images (A) Preoperative and (B and C) postoperative images.

A three-year-old Lebanese girl was brought by her parents with concerns regarding the appearance of her feet and associated difficulty walking. Clinical examination revealed a single central ray deficiency in the right foot and three central ray deficiencies in the left one. There was also a complete syndactyly between the fourth and fifth toes in the left foot, along with a hallux valgus deformity. The review of systems was unremarkable, and the child denied any pain. Family history was negative for similar conditions or other congenital anomalies. The patient underwent surgical correction of her left cleft foot with pre- and postoperative images shown in Figure 1.

**Figure 2:**
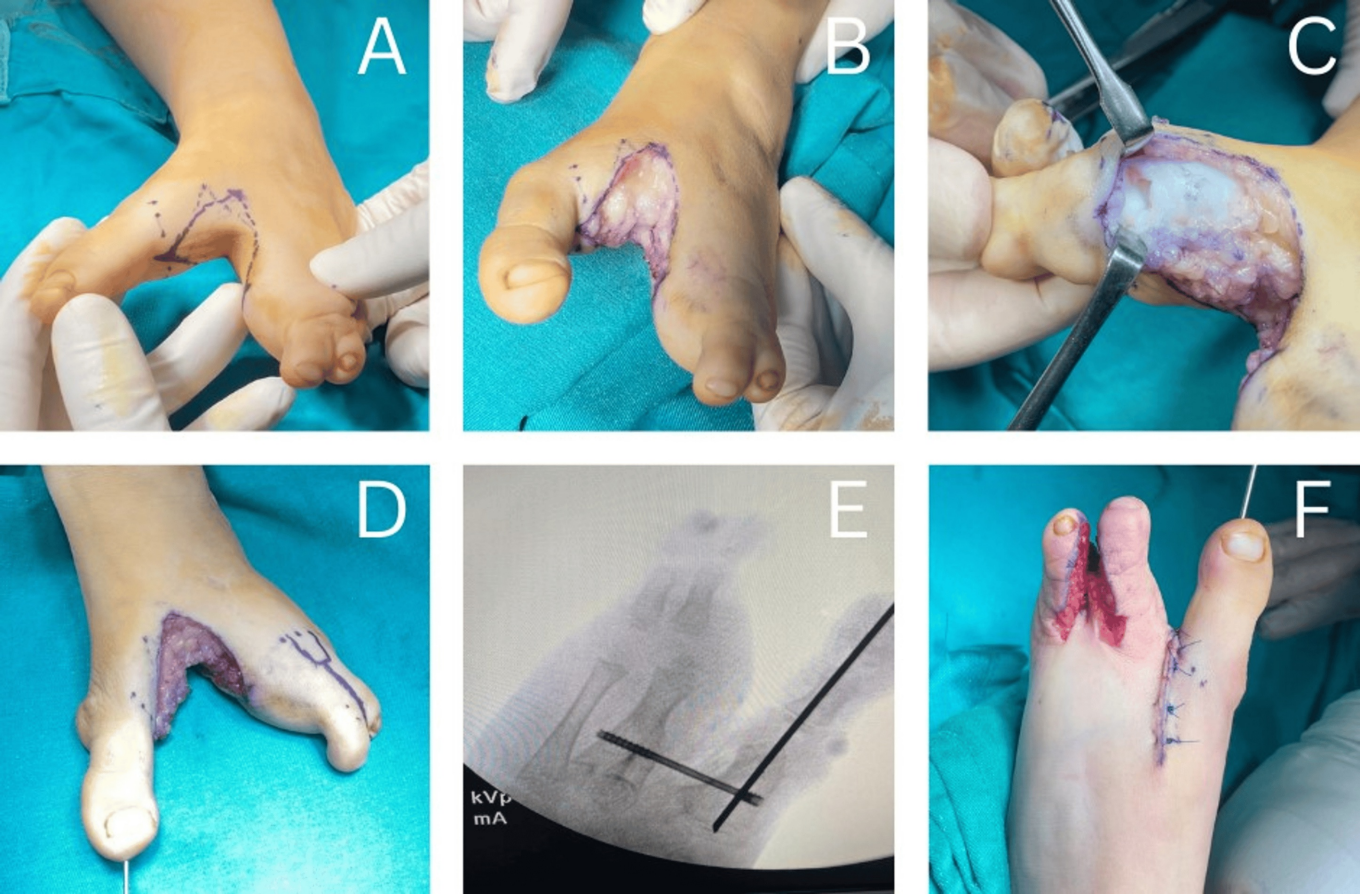
Sequential steps of the surgical technique (A) Margins of the cleft were outlined with a sterile ink marker on the dorsum, sole and both sides, defining the planned excision. (B) The cleft was opened along the markings, creating a rhomboid-shaped defect. (C) Capsular release of the interphalangeal joint. (D) Intramedullary K-wire fixation of the first ray for valgus deformity correction. (E) Cannulated screw fixation on the adjacent metatarsals to reduce forefoot width. (F) Syndactyly release of the fourth web space.

Figures 2A-2F illustrate the sequential steps of the surgical technique in five labeled images, each accompanied by an explanation. The cleft was first manually approximated by stabilizing the foot with one hand, and a sterile ink marker was used to outline the cleft’s margins on both the dorsal and plantar surfaces, defining the planned lines for skin incision (Figure 2A). The cleft was then opened along these markings, creating the diamond-shaped area of excised skin (Figure 2B). Throughout the procedure, special care was taken to preserve the neurovascular structures while excising the skin and subcutaneous tissue.

After flap elevation, correction of the hallux valgus deformity was performed. Capsular release of the interphalangeal joints was carried out, along with intramedullary K-wire fixation of the first ray (Figures 2C-2D). Subsequently, a cannulated screw was inserted through drilled holes in the adjacent metatarsals (first and fourth metatarsal heads) to reduce the intermetatarsal distance and narrow the forefoot (Figure 2E).

Once hemostasis was achieved, the flaps were repositioned. During the same procedure, complete syndactyly in the left fourth web space was corrected (Figure 2F). The resulting defect was reconstructed with a full-thickness skin graft taken from the excised diamond-shaped tissue. Postoperatively, splints were applied for six weeks, after which the patient was allowed to walk. The procedure was performed under general anesthesia, and the postoperative course was uneventful, with proper wound healing and no complications. Functional success was defined by the ability to ambulate and wear standard footwear, eliminating the need for custom-made shoes. Aesthetically, a well-contoured foot was achieved through the diamond-shaped incision technique. At a one-year follow-up, the patient demonstrated full, pain-free ambulation, was able to wear regular footwear, and participate in daily activities without limitation. The range of motion of the remaining toes was preserved, and the correction was maintained. Management of the right foot was considered but ultimately declined by the family for financial reasons.

## Discussion

Cleft foot is a rare congenital malformation presenting with varying degrees of foot structural deficiency. The primary goals of surgical intervention are to restore standard footwear and improve the overall cosmetic appearance of the foot. Given the wide range of severity in cleft-foot deformities, there is no single standardized surgical procedure; instead, treatment must be customized based on the specific characteristics of each case [5-7].

Classification systems and contextualization of the present case

**Figure 3:**
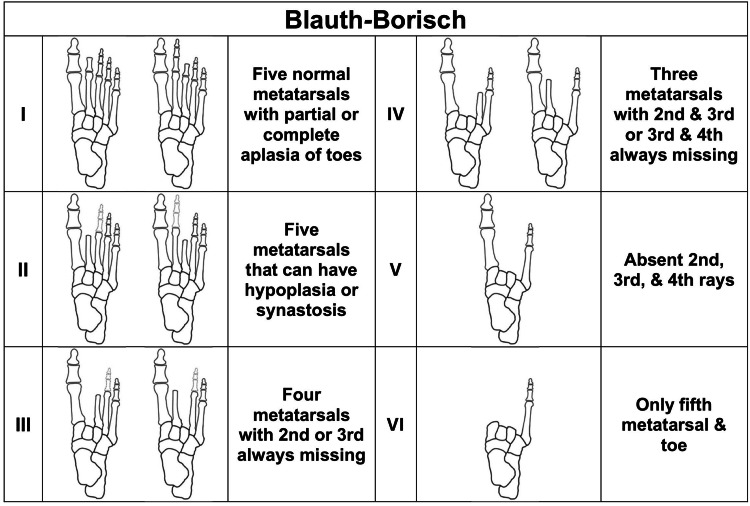
Schematic illustration and descriptive overview of the Blauth-Borisch classification for cleft foot Attenuated structures represent anatomical elements that may or may not be available. Image credit: [3]

The diverse presentation of cleft foot necessitates a structured classification system to accurately describe the deformity and guide the surgical approach. In 1990, Blauth and Borisch analyzed radiographic findings and features of 173 feet and proposed a system that categorizes cleft foot deformities into six distinct types based on the number of metatarsal bones present, as shown in Figure 3 [3].

In 1999, Abraham et al. proposed a refined clinical classification system that serves as a framework for guiding treatment decisions. Type I is characterized by the absence of the second or third ray extending to the level of the metatarsals; management typically involves creating syndactyly between the remaining rays, with correction of any associated hallux valgus deformity, if needed. Type II is defined by a deep cleft extending to the tarsal bones and involving the forefoot; treatment includes creating syndactyly and performing an osteotomy of the first ray. However, in patients older than five years, amputation of the first ray is generally recommended. Type III is identified by the complete absence of the first through third or fourth rays, for which surgical intervention is usually not required. Based on this classification, treatment was performed on 32 feet of 16 patients, with satisfactory outcomes in 23 out of the 24 procedures reported [5].

Our patient presented with a cleft foot classified as Blauth and Borisch Type III and Abraham Type II. The complexity of this mixed presentation necessitated a highly customized surgical approach that would simultaneously address metatarsal alignment, forefoot narrowing, and tension-free soft-tissue closure.

Surgical techniques in literature

Management strategies for cleft foot can vary from simple observation in mild cases to surgical procedures aimed at closing the cleft while preserving the toes. In more complex cases, surgical intervention may involve the removal of affected toes when necessary or reconstructive techniques to restore missing structures [4]. The choice of treatment depends on the extent of the malformation to ensure optimal functional and aesthetic outcomes for each patient [6]. To address these concerns, a literature review was conducted to summarize reported surgical techniques, highlighting flap design and bony correction with fixation methods.

Several techniques have been described to address the soft-tissue and flap component of the deformity. Sumiya and Onizuka (1980) aimed for a five-toe reconstruction using double pedicle flaps harvested from the cleft region, achieving outstanding outcomes in both functional and aesthetic aspects [8]. Subsequently, Takagi and Kadomatsu (2019) proposed a novel flap-bag technique combining a plantar triangular and dorsal rectangular flap to yield better outcomes by decreasing scarring and maintaining natural foot space [9]. More recently, Rout et al. (2024) reported successful reconstruction of a near-normal first web space using a zigzag incision [2]. On the other hand, our technique employed a diamond-shaped incision with a rotational flap design to achieve a secure soft-tissue closure while preserving the neurovascular bundles.

In addition to soft-tissue reconstruction, numerous successful techniques in the literature emphasize closure of the bony gap and stabilization of the corrected alignment. Lejman and Michno (1998) performed wedge resection of the metatarsals with screw fixation, followed by skin syndactylization, reporting good patient satisfaction [10]. Another successful technique was observed by Wood et al. (1997), who incorporated triangular flaps along with metatarsal osteotomies, reconstruction of the intermetatarsal ligament, and stabilization using Kirschner wires [7]. Choudry et al. (2010) reported a similar approach applied to five feet of three patients, using a central wedge excision and osteotomy of the fifth metatarsal, followed by K-wire and soft-tissue syndactylization, with good long-term functional and cosmetic results [11]. Sunagawa et al. (2002) described a microvascular transfer of a toe from the amputated contralateral side, with the grafted toe exhibiting symmetric longitudinal growth compared to the other toes [12]. Talusan et al. (2013) and Narang et al. (2021) employed a similar suture-button fixation technique to approximate the adjacent metatarsals and achieve cleft closure, with Talusan et al. (2013) emphasizing the intermetatarsal ligament reconstruction using a TightRope system and Narang et al. (2021) applying the method through a fish-mouth incision for cosmetic and functional correction [1,13]. Lastly, Leonchuk et al. (2020) proposed wire fixation and realignment using the Ilizarov apparatus [4]. In our case, gap stabilization and alignment were performed using a cannulated screw between the first and fourth metatarsals to narrow the intermetatarsal gaps, supplemented by K-wire fixation to maintain alignment and ensure stability during healing.

Management of our case followed a similar approach to that of Ergün and Öztürk [6], but with key modifications. A diamond incision was used instead of a Z-plasty to achieve better contour and tension-free closure, and a cannulated screw replaced the Ethibond to provide rigid fixation of the metatarsals. Additionally, our case involved a more severe classification, necessitating greater correction and meticulous soft-tissue management. These modifications allowed for a more stable construct and enabled tighter, more controlled closure of the intermetatarsal gap.

Limitations of modified technique and alternative management

The modified technique proposed in this case, combining a diamond-shaped incision with fixation using both K-wires and cannulated screws, yielded promising results. At the one-year follow-up, the patient was able to walk independently, wearing off-the-shelf footwear without restrictions. Although no validated scoring system exists for functional assessment of cleft foot, the patient’s recovery was assessed descriptively and considered excellent, meeting our two goals set primarily: function and appearance.

Despite these positive outcomes, this surgical approach carries several limitations. The approach requires high technical precision to insert the cannulated screw through the adjacent metatarsals while preserving soft tissue from any further damage and avoiding joint surfaces. This increases operative time and necessitates intraoperative imaging for accurate alignment. Furthermore, as with all K-wire procedures, there remains an inherent risk of infection, requiring strict aseptic technique and careful post-operative monitoring. Additionally, subsequent minor surgeries may be required to remove cannulated screws, posing an extra burden for the patient and family. Finally, soft-tissue reconstruction requires precise flap design to ensure tension-free, well-perfused closure, given the limited availability of healthy tissue.

Non-surgical management may be considered in very mild, asymptomatic cases [4]. Conservative options include specialized footwear designed to distribute the pressure and provide forefoot stability; nonetheless, these approaches don’t correct the underlying deformity [14]. Tani et al. (2000) demonstrated that, while simple closure is effective in mild cases with no central ray deficiency, achieving satisfactory results becomes more challenging when two or three central rays are missing [15]. Currently, there is no validated protocol or outcome data addressing orthotics, adaptive footwear, or observation for this condition, with most of the evidence being largely descriptive. Therefore, surgical intervention remains the mainstream of treatment for most cleft foot cases to ensure long-term functional and aesthetic outcomes.

This report provides valuable insight into the existing literature on cleft foot; however, it is not without limitations. Being a single case report, its findings are inherently limited in generalizability and may be subject to selection bias. Moreover, functional recovery and quantitative outcomes were restricted to descriptive objectives. Finally, although surgery was uneventful, longer follow-up and further studies involving multiple cases are needed to confirm durability and reproducibility.

## Conclusions

In conclusion, this case report illustrates the surgical technique used in the successful management of a rare bilateral cleft foot deformity (Blauth and Borisch Type III and Abraham Type II) in a pediatric patient. Using a diamond-shaped incision with cannulated screw fixation and K-wire achieved an aesthetically well-contoured outcome, and the patient was able to walk freely and comfortably at one-year follow-up. The results highlight the importance of a tailored surgical approach for the variably complex deformities of cleft foot. Furthermore, this report contributes to the growing body of evidence supporting integrated surgical interventions that address both function and cosmetic appearance. Further extensive follow-up is essential to assess the long-term durability of this technique. Given the rarity of this condition, future efforts should focus on retrospective multicenter analysis and scoping reviews of published cases. Such work would integrate scattered clinical experience, highlight effective surgical approaches across diverse presentations, and contribute to the formulation of evidence-based management guidelines.
